# Data on myeloperoxidase-oxidized low-density lipoproteins stimulation of cells to induce release of resolvin-D1

**DOI:** 10.1016/j.dib.2018.03.131

**Published:** 2018-04-04

**Authors:** Damien Dufour, Alia Khalil, Vincent Nuyens, Alexandre Rousseau, Cédric Delporte, Caroline Noyon, Melissa Cortese, Florence Reyé, Valérie Pireaux, Jean Nève, Luc Vanhamme, Bernard Robaye, Christophe Lelubre, Jean-Marc Desmet, Martine Raes, Karim Zouaoui Boudjeltia, Pierre Van Antwerpen

**Affiliations:** a Laboratory of Pharmaceutical Chemistry and Analytical Platform of the Faculty of Pharmacy, Faculty of Pharmacy, Université Libre de Bruxelles, Brussels, Belgium; b Laboratory of Experimental Medicine (ULB 222 Unit), CHU de Charleroi, A. Vésale Hospital, Université Libre de Bruxelles, Montigny-le-Tilleul, Belgium; c Laboratory of Neurovascular Signaling, Universite libre de Bruxelles, Gosselies, Belgium; d URBC-Narilis, University of Namur, 5000 Namur, Belgium; e Unit of Dialysis, CHU de Charleroi, A. Vésale Hospital, Université Libre de Bruxelles, Montigny-le-Tilleul, Belgium; f Institute of Interdisciplinary Research, IRIBHM, Universite Libre de Bruxelles, Gosselies, Belgium

## Abstract

This article present data related to the publication entitled “Native and myeloperoxidase-oxidized low-density lipoproteins act in synergy to induce release of resolvin-D1 from endothelial cells” (Dufour et al., 2018). The supporting materials include results obtained by Mox-LDLs stimulated macrophages and investigation performed on scavenger receptors. Linear regressions (RvD1 vs age of mice and RvD1 vs CL-Tyr/Tyr) and Data related to validation were also presented. The interpretation of these data and further extensive insights can be found in Dufour et al. (2018) [1].

**Specifications table**

**Table:** 

Subject area	Biochemistry
More specific subject area	Resolvin D1 in atherosclerosis
Type of data	Table, text file, graph, figure
How data was acquired	Mass spectrometry (LC-MS-/-MS system from Agilent Technologies (Santa Clara, CA, USA): an Agilent 1290 Infinity Binary - UHPLC system coupled to a mass spectrometer Agilent Jet Stream electrospray ionization source (AJS)-Triple Quadrupole (QQQ) 6490 series)
	Rt-PCR
Data format	Analyzed
Experimental factors	Samples were treated by liquid-liquid extraction before analysis
Experimental features	RvD1 and precursors (17S-HDHA and DHA) were quantified in cell supernatant or in plasma.
Data source location	Brussels, Belgium
Data accessibility	The data are provided with this article

**Value of the data**

• Validation of method was an important part of this work and we showed how it was developed.
• Data show how scavenger receptors, usually involved in oxidized LDLs recognition, were analyzed and how they could be involved in RvD1 synthesis.
• Correlation was established between level of RvD1 and Cl-Tyr/Tyr ratio from healthy donors and between level of RvD1 and age in mice. These results are illustrated here.
• Because HMEC have shown ability to produce RvD1, we assay the production of RvD1 by monocytes (THP-1).

## 1 Data

Different aspects of method validation for RvD1 and precursors analysis were described. Moreover, we investigated many aspects of RvD1 mechanistic of production linked to Mox-LDLs stimulation of endothelial cells and THP-1 macrophages.

### 1.1 Macrophages are apparently not essential for RvD1 production

**Fig. 1:**
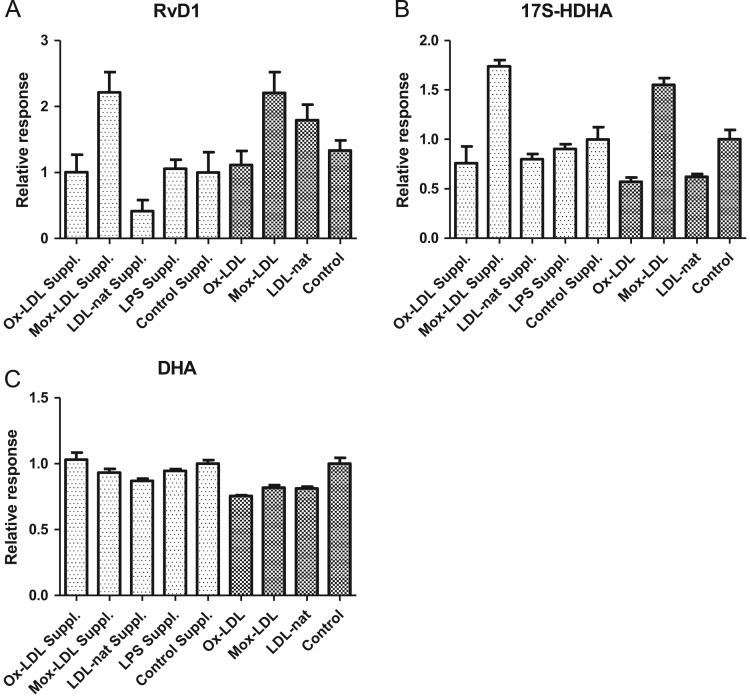
(A), (B), (C): Incubation of THP-1: Data for RvD1 (A), 17S-HDHA (B) and DHA (C) after 24 h of incubation of THP-1 with Ox-LDLs, Mox-LDLs or LDLs-nat with (light grey bars) or without (dark grey bars) supplementation of 10 ng/ml 17S-HDHA and DHA. Data are expressed as mean ± SD of four independent experiments compared to the average of four controls (control RvD1: 0.02 ± 0.01 ng/ml; 17S-HDHA: 0.26 ± 0.05 ng/ml; DHA: 21 ± 2 ng/ml).

It is generally accepted that EC and monocytes/macrophages are both required to produce RvD1. However, it was shown that EC are able to produce RvD1 alone [1]. Therefore, we tested whether Mox-LDLs induced RvD1 production in the presence of THP-1. Cells were incubated with 100 μg/ml of Mox-LDLs and data showed an increased concentration of RvD1 (76 ± 21 pg/ml) compared with THP-1 cells incubated with 100 μg/ml non-physiologic Ox-LDLs (RvD1 35 ± 16 pg/ml) or 100 μg/ml LDLs-nat (RvD1 14 ± 10 pg/ml). However, the differences were not statistically significant. An increase in 17S-HDHA was also observed in the same conditions with a concentration of 439 ± 32 pg/ml when incubated with 100 μg/ml of Mox-LDLs and concentrations of 192 ± 84 pg/ml and 202 ± 25 pg/ml when incubated with Ox-LDLs or LDLs-nat, respectively. These data were again not statistically significant (see Fig. 1).

## 2 Experimental design, materials and methods

### 2.1 Macrophages are apparently not essential for RvD1 production

**Fig. 2:**
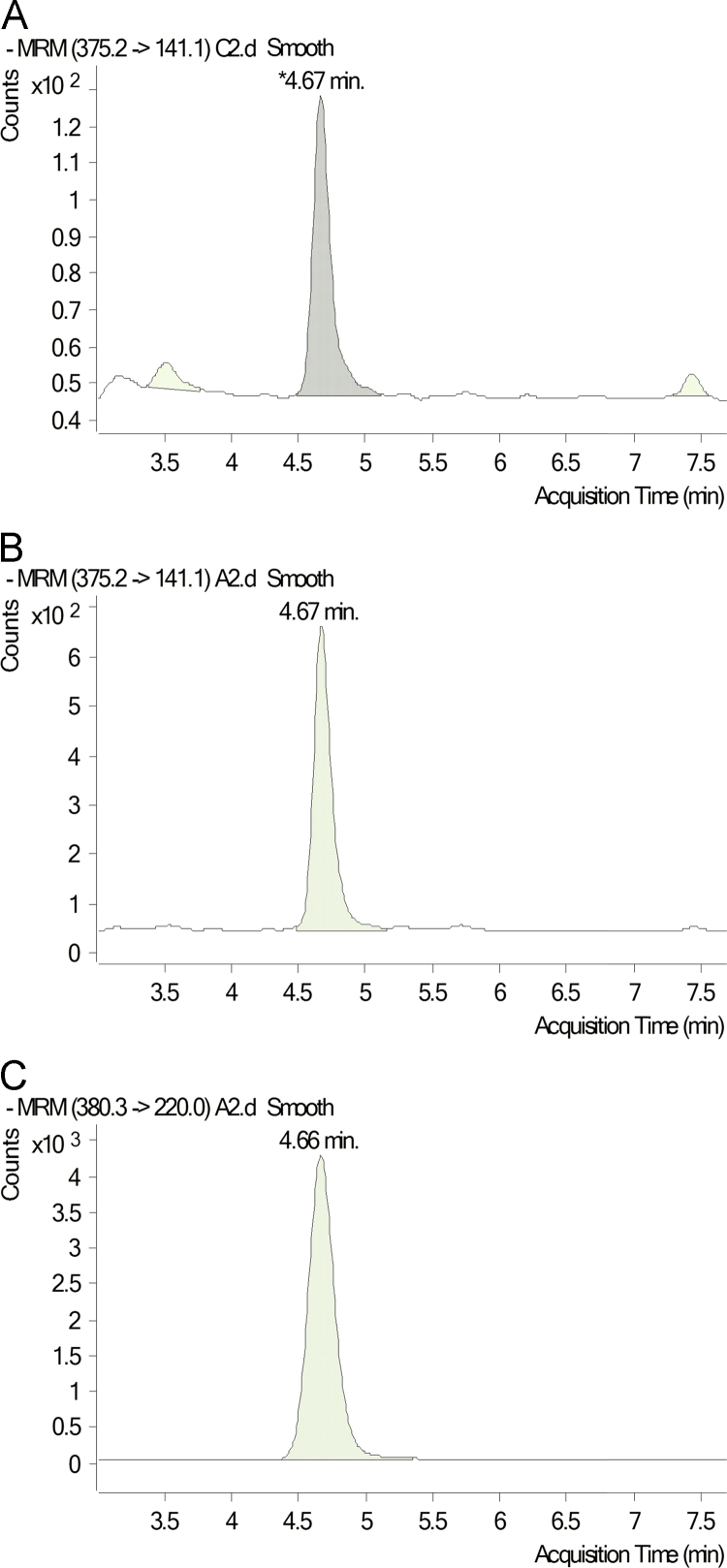
(A), (B), (C): Chromatograms obtained by LC/MS-MS analysis of RvD1 from plasma of healthy donor without supplementation (A) or spiked by 0.5 ng/ml of standard (B) and compared to Internal standard RvD1-d5 (C).

**Fig. 3:**
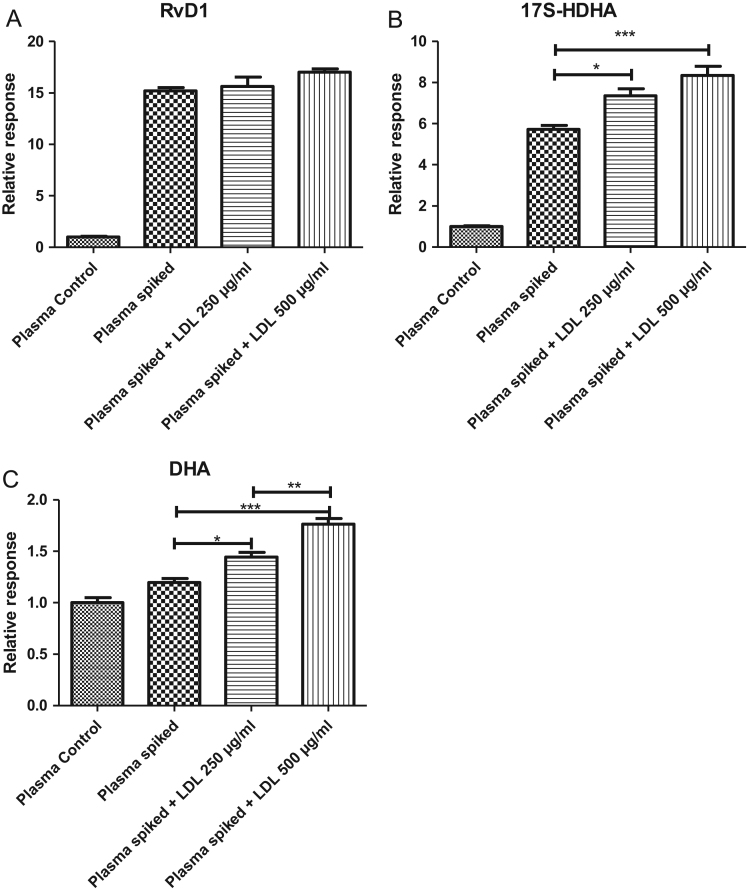
(A), (B), (C): Effect of lipeamia on quantification of RvD1, 17S-HDHA and DHA: Quantification of RvD1 (A), 17S-HDHA (B) and DHA (C) in sample of plasma from healthy donor, spiked by RvD1, 17S-HDHA and DHA (0.5 ng/ml, 5 ng/ml and 100 ng/ml respectively) and by different concentration of LDLs-nat (0, 250, 500 µg/ml). Data are expressed as mean ± SD of four independent experiments compared to the average of four controls obtained with by plasma not spiked (control RvD1: 0.03 ± 0.01 ng/ml; 17S-HDHA: 0.62 ± 0.04 ng/ml; DHA: 566 ± 48 ng/ml). * *p* < 0.05; ** *p* < 0.01 and *** *p* < 0.001 by Tukey Test.

**Fig. 4:**
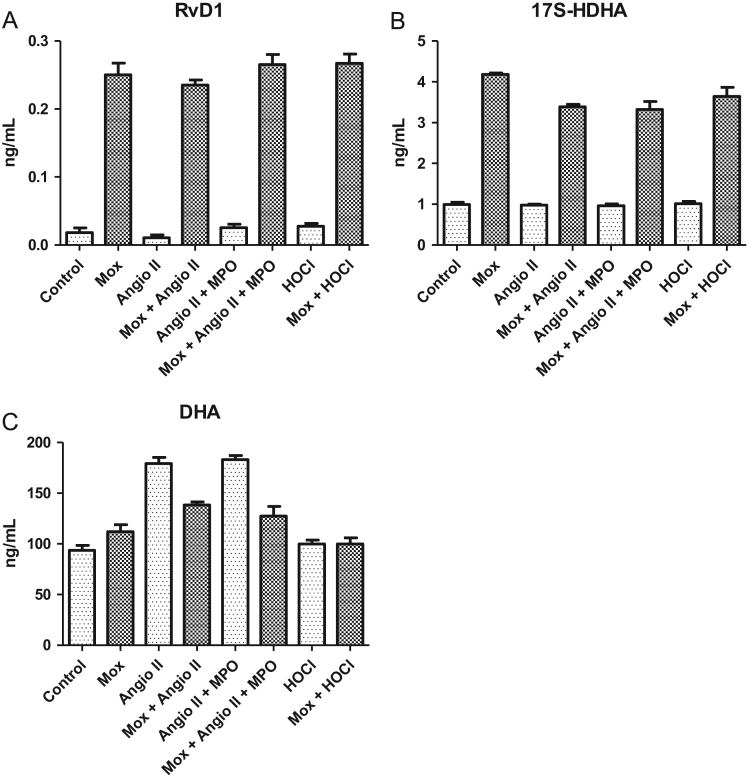
(A), (B), (C): RvD1 by MPO system stimulation of HMEC: concentrations (ng/ml) obtained for RvD1 (A), 17S-HDHA (B) and DHA (C) after stimulation of HMEC by MPO system, Angiotensine II, Mox-LDLs or HOCl.

**Fig. 5:**
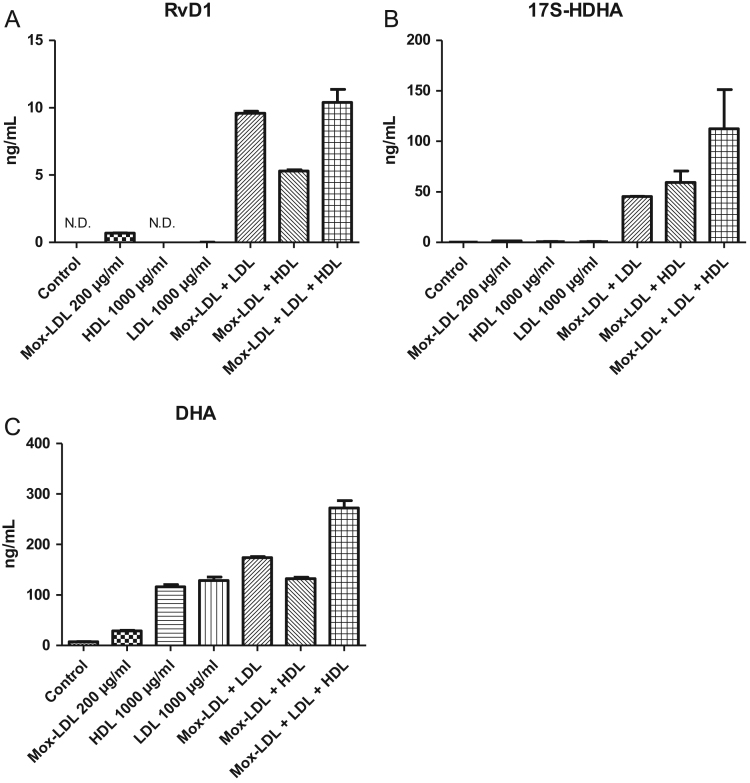
(A), (B), (C): Effect of LDLs-nat, HDLs and Mox-LDLs on HMEC: Quantification of RvD1 (A), 17S-HDHA (B) and DHA (C) in supernatants of cells stimulated by LDLs-nat (1000 µg/ml), HDLs (1000 µg/ml) and Mox-LDLs (200 µg/ml) for 24 h at 37 °C. Data are expressed as mean ± SD of four independent experiments. Control was carried out on by HMEC alone.

**Fig. 6:**
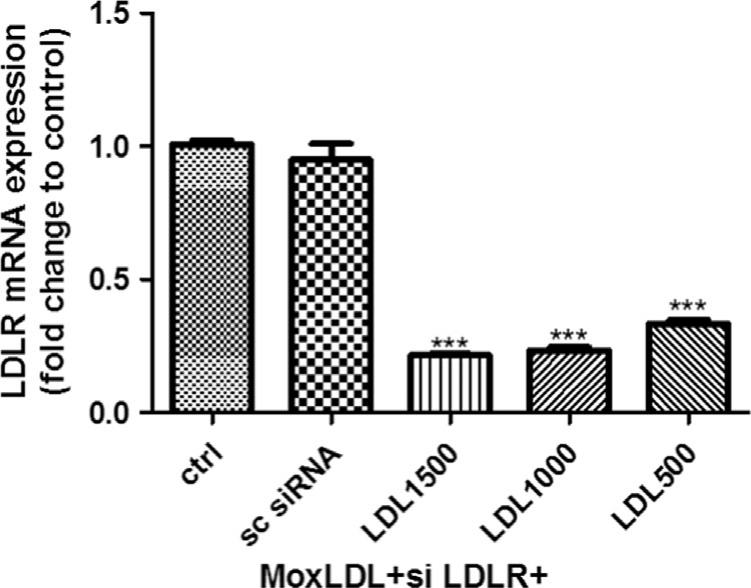
Validation of LDLR gene silencing efficiency using siRNA: RNA was extracted and analyzed by qRT-PCR. Expression levels were normalized to GAPDH. Values are expressed as fold change compared to the control. Data were evaluated by One-Way ANOVA test * *p* < 0.05.

**Fig. 7:**
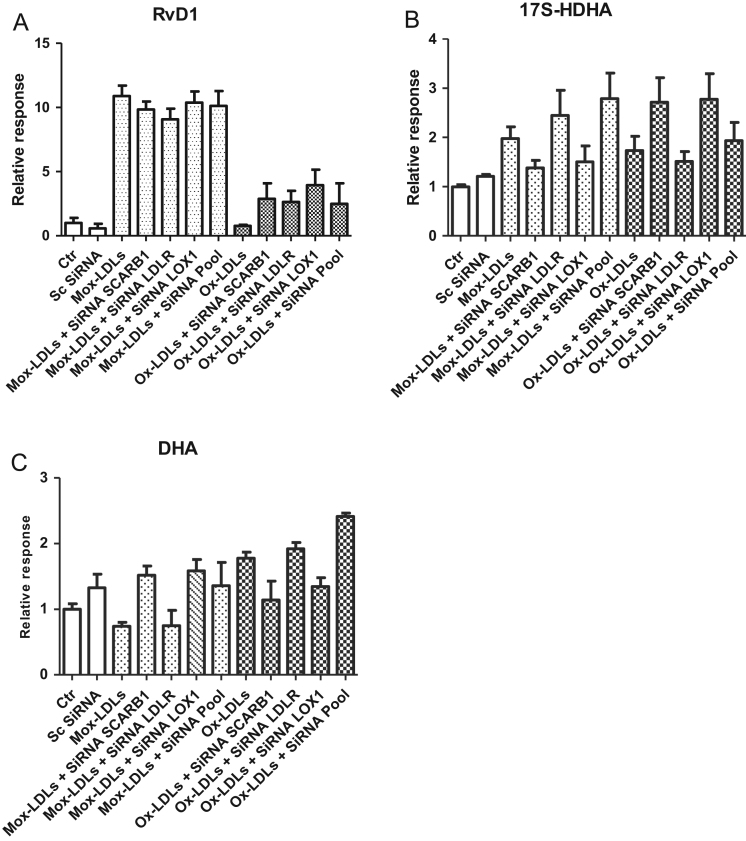
(A), (B), (C): Control of integration of Mox-LDL or Ox-LDL by LDL receptor: Relative response of RvD1 (A), 17S-HDHA (B) and DHA (C) calculated after incubation of endothelial cells with SiRNA LDLr, SiRNA SCARB1, SiRNA LOX-1 or all SiRNA pooled in presence of Mox-LDL or Ox-LDL (100 µg/ml). Controls were realized by endothelial cells without transection or transfected by scrambled siRNA.

**Fig. 8:**
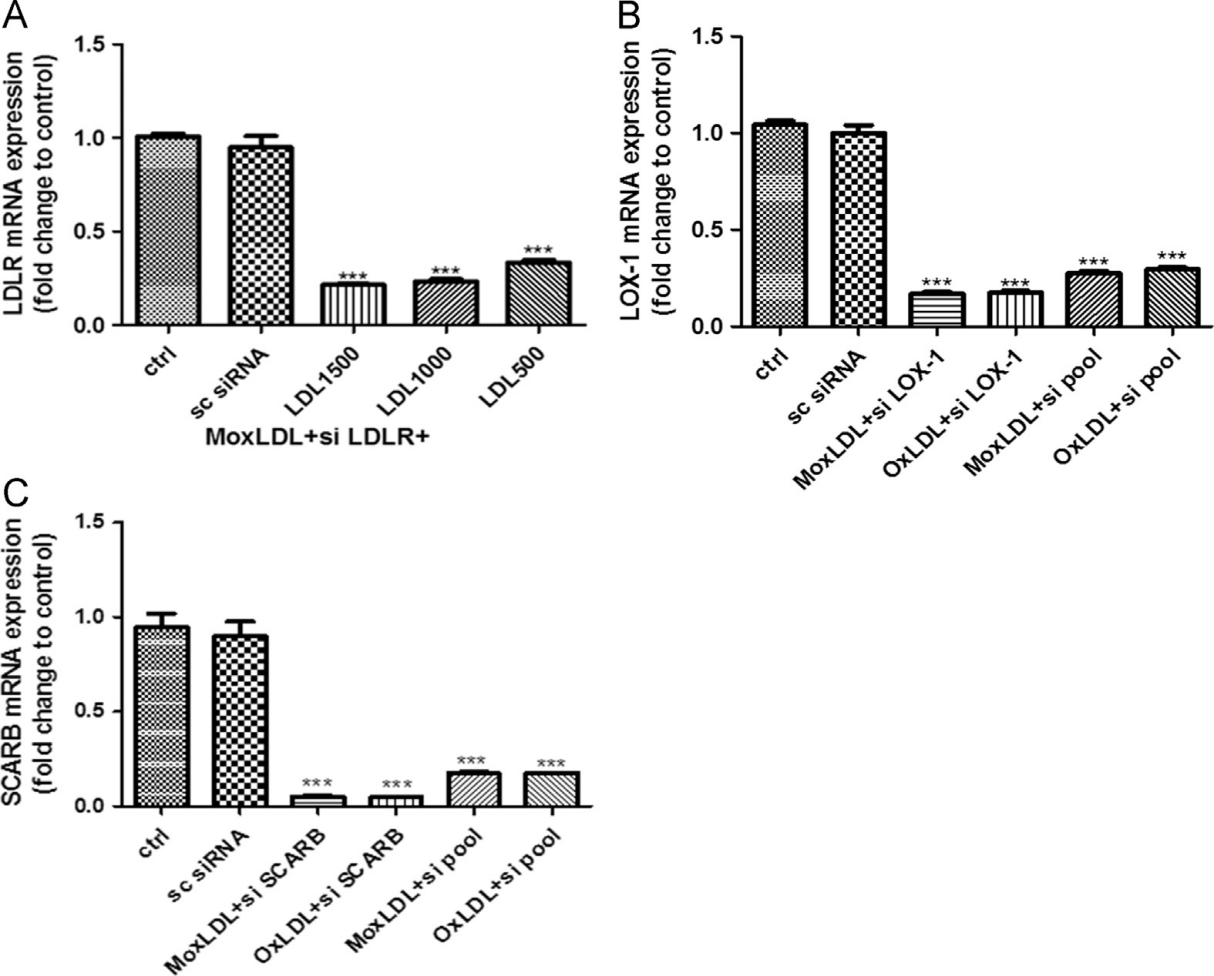
(A), (B), (C): Validation of gene silencing efficiency using siRNA: HMEC-1 cells were transfected with scrambled siRNA (sc siRNA) or LDLR siRNA for 48 h then treated with Mox-LDL or OxLDL (100 µg/ml) for another 24 h. RNa was then extracted and analyzed by qRT-PCR using primers specific for LDLR. The expression level was normalized to GAPDH. Values are expressed as the fold change to the control. Data were evaluated by one-way ANOVA test: * *p* < 0.05, ** *p* < 0.01 and *** *p* < 0.001 vs control were considered significant.

**Fig. 9:**
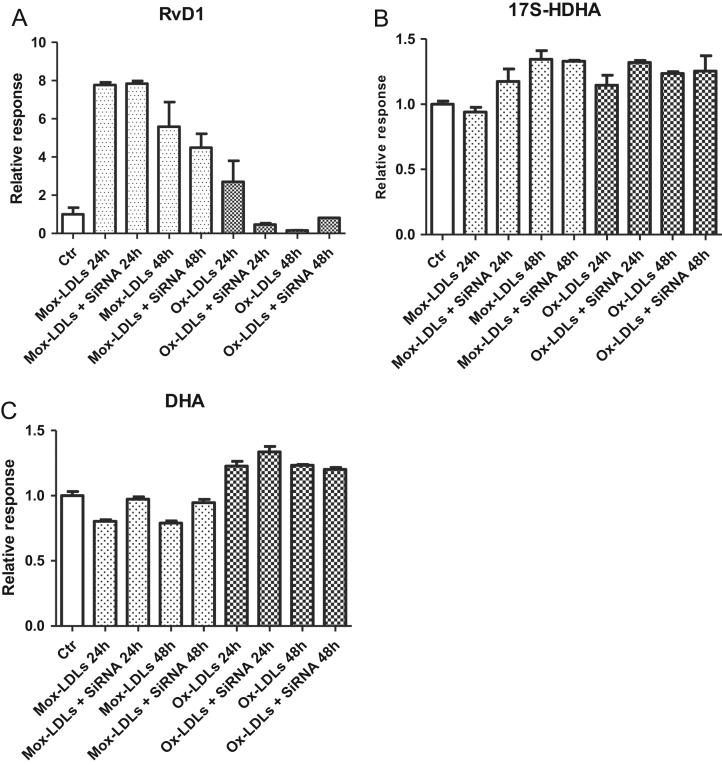
(A), (B), (C): Effect of time on SiRNA LOX-1: Relative intensity of RvD1 (A), 17S-HDHA (B) and DHA (C) calculated by normalization to control after incubation of endothelial cells with SiRNA LOX-1 in presence of Mox-LDL or Ox-LDL (100 µg/ml) for 24 h or 48 h. Controls were realized by endothelial cells without transection.

**Fig. 10:**
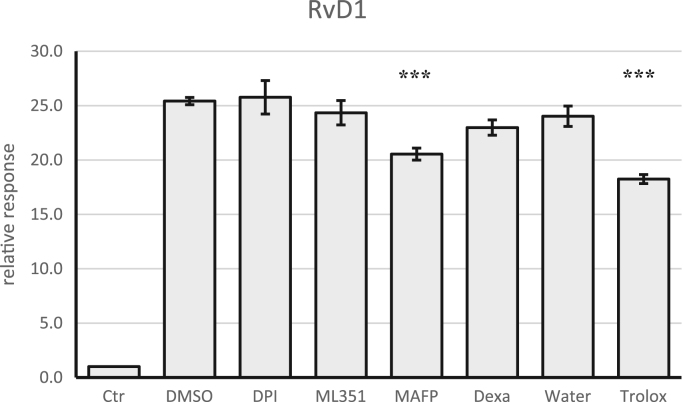
Quantification of RvD1 after 24 h of incubation of endothelial cells with DMSO (vehicle), DPI (NADHP oxidase and NOS inhibitor), ML351 (lipoxygenase inhibitor), MAFPF (PLA_2_ inhibitor), dexamethasone (anti-inflammatory), water (vehicle) Trolox (antioxidant) in presence of Mox-LDL compared to a control. Data are given as relative response normalized of 4 independent experiments. Data were considered as significant (MAFP vs DMSO and Trolox vs Water) by ANOVA: *p* < 0.001 (***).

**Fig. 11:**
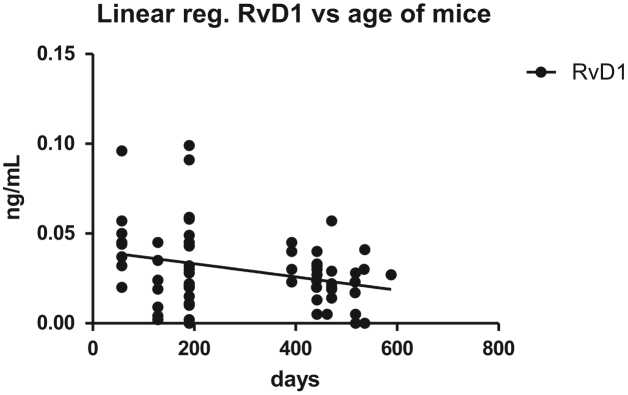
Linear regression of RvD1 versus age of mice s (*n* = 65, *♀* = 40, *♂*= 25, middle age = 291 ± 270 days, *R*^2^ = 0.08782, *p* = 0.0165, slope = −0.00006673 to −0.000006936).

**Fig. 12:**
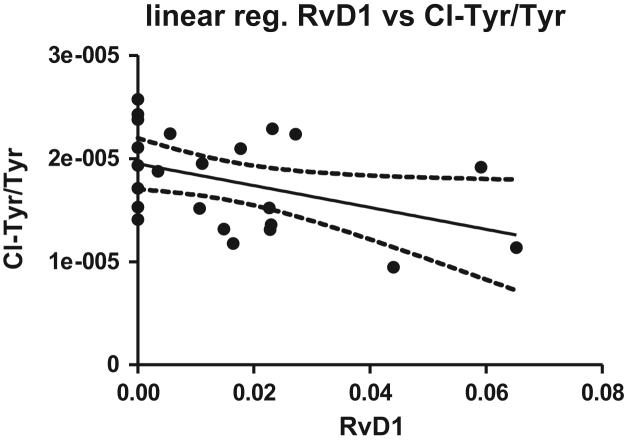
Linear regression of RvD1 versus ratio Chloro-tyrosine/tyrosine from healthy donors (*n* = 23, *R*^2^ = 0.1809, *p* = 0.0430, slope = −0.0001060 ± 0.00004921).

Because we suspected that THP-1 may participate in RvD1 production, we also tested the effects of Mox-LDLs, non-physiologic Ox-LDLs and LDLs-nat on the synthesis of RvD1 and 17S-HDHA by THP-1. THP-1 were incubated with 200 μg/ml of Mox-LDLs, LDLs-nat or non-physiologic Ox-LDLs. The effect of supplementation with 10 ng/ml of DHA and 17S-HDHA was also tested. Samples were purified by the previous method and RvD1, 17S-HDHA and DHA were quantified using LC-MS/MS as illustrated in Fig. 1 (Fig. 2, Fig. 3, Fig. 4, Fig. 5, Fig. 6, Fig. 7, Fig. 8, Fig. 9, Fig. 10, Fig. 11, Fig. 12). The quantification of RvD1 and precursors was tested in the presence of different LDL concentrations (see Fig 2). For details on the method validation, see Dufour et al. (2018) and tables 1 and 2. This method was used for quantification of RvD1 and precursors in different matrices like cells supernatant and after genesilencing or plasma (see Fig. 3, Fig. 4, Fig. 5, Fig. 6, Fig. 7, Fig. 8, Fig. 9, Fig. 10, Fig. 11, Fig. 12). See Dufour et al. (2018) [1] for more information.

### 2.2 in vivo macrophage subpopulations analysis using flow cytometry

100 µL of total blood were incubated for 15 min at RT with PE mouse anti-human CD14 and V500 mouse anti-human CD16 antibodies (Becton Dickinson, Franklin Lakes, NJ, USA) as well as with mouse anti-human CD86-FITC and anti-human CCR2-APC monoclonal antibodies (Miltenyi Biotec, Bergisch Gladbach, Germany) for determining the M1 polarization or with mouse anti-human CD206-FITC, anti-human CXCR3-APC and anti-human CD163-VioBlue monoclonal antibodies (Miltenyi Biotec, Bergisch Gladbach, Germany) for the M2 polarization. Red blood cells were then eliminated by adding BD FACS Lysing Solution (dilution: 1/20) (Becton Dickinson, Franklin Lakes, NJ, USA) to total blood and remaining cells were washed twice with 1 mL of running buffer. Cells were finally resuspended in 300 µL of running buffer for analysis. The matching isotype controls were used for each antibody in order to define the threshold. The analysis was performed using the MACSQuant Analyzer 10 (Miltenyi Biotec, Bergisch Gladbach, Germany), applying a gating strategy based on the SSC vs PE gate (CD14), selecting the monocyte population. Classical monocytes were defined based on a high expression of CD14 and a low expression of CD16 (CD14+CD16-). Intermediate monocytes were defined based on a high expression of CD14 and CD16 (CD14+CD16+), while non-classical monocytes were characterized by a low expression of CD14 and a high expression of CD16 (CD14-CD16+) (Table 1, Table 2, Table 3).

**Table 1:** Transitions optimization: Transitions associated to collision energy used like quantifier or qualifier and retention time in minutes for each compound. These transitions were obtained using Optimizer program from Agilent Technologies and were confirmed by manual optimization.

Compounds	(M-H)^-^	Quantifier m/z (coll. Energy)	Qualifier m/z (coll. Energy)	Retention time (min)
RvD1	375.22	141.1 (16 eV)	215.1 (16 eV)	4.8
RvD1-d5	380.25	141.1 (13 eV)	220 (17 eV)	4.8
17S-HDHA	343.23	281.3 (8 eV)	325.3 (8 eV)	7.0
PGE2-d4	355.2	319.1 (4 eV)	275.2 (12 eV)	4.5
DHA	327.23	283.2 (8 eV)	58.9 (40 eV)	8.2
DHA-d5	332.26	288.0 (5 eV)	234.3 (9 eV)	8.2

**Table 2:** Data of validation: Recovery and coefficients of variation (CV) obtained using liquid/liquid purification of plasma spiked with two different concentrations of RvD1 (1 ng/ml and 0.5 ng/ml), 17S-HDHA (10 ng/ml and 5 ng/ml) and DHA (200 ng/ml and 100 ng/ml).

Product	Concentration (ng/ml)	Recovery (%)	CV (%)
RvD1	1	86.1 ± 8.1	9.4
	0.5	90.2 ± 3.1	3.4
			
17S-HDHA	10	45.2 ± 12.0	26.5
	5	53.0 ± 7.7	14.6
			
DHA	200	100.1 ± 13.2	13.2
	100	104.5 ± 5.4	5.2

**Table 3:** List of primers: primers used for qRT-PCR.

Gene	Reverse sequence (5’-3’)	Forward sequence (5’-3’)
OLR1	CGTGACTGCTTCACTCTCTCAT	TCAGACACCTGGGATAATTGCAT
LDLr	CCCTGACGAATTCCAGTGCT	GAGTGTCACATTAACGCAGCC
SCARB1	GGCCATTCAGGCCTATTCTGA	TCCTCAGGACCCTACAGTTTTG
GAPDH	ACCCACTCCTCCACCTTTGAC	GTCCACCACCCTGTTGCTGTA
