# Supplementary material for: Data on myeloperoxidase-oxidized low-density lipoproteins stimulation of cells to induce release of resolvin-D1
Source: Data Brief. 2018 Apr 4;18:1160–71. doi: 10.1016/j.dib.2018.03.131 (PMC5996617; doi:10.1016/j.dib.2018.03.131)
Supplement: Supplementary file 1 — Supplementary material [file mmc1.docx]

Conflict of Interest

The author declares as no conflict of interest.
